# SARS-CoV-2 Infection and Risk Management in Multiple Sclerosis

**DOI:** 10.3390/diseases9020032

**Published:** 2021-04-19

**Authors:** Amado Diaz de la Fe, Alejandro Armando Peláez Suárez, Marinet Fuentes Campos, Maivis Noemí Cabrera Hernández, Carlos-Alberto Goncalves, Stephen Schultz, Dario Siniscalco, Maria Angeles Robinson-Agramonte

**Affiliations:** 1 Neuromuscular Diseases Clinic, International Center for Neurological Restoration, Habana 11300, Cuba; amado@neuro.ciren.cu (A.D.d.l.F.); pelaezsuarezalejandro@gmail.com (A.A.P.S.); 2 Departamento de Medicina Familiar y Comunitaria Policlínico 28 de Enero, Habana 11300, Cuba; marinetfuentes@gmail.com; 3 Consulta de Alergia, Hospital General Secretaria de Salud, Iguala City 40000, Mexico; maivis8@hotmail.com; 4 Department of Biochemistry, Federal University of Rio Grande do Sul, Porto Alegre 90040-060, Brazil; casg@ufrgs.br; 5 Department of Cellular and Integrative Physiology, Center for Biomedical Neuroscience, University of Texas (UT) Health Science Center San Antonio, San Antonio, TX 78229, USA; stevendri0629@gmail.com; 6 Department of Experimental Medicine, University of Campania, 80138 Naples, Italy; dario.siniscalco@unicampania.it; 7 Neuroimmunology Department, Research Center, International Center for Neurological Restoration, Habana 11300, Cuba

**Keywords:** SARS-CoV-2, COVID-19, multiple sclerosis (MS), benefit versus risk in multiple sclerosis (BRMS)

## Abstract

The novel coronavirus can cause a severe respiratory disease with impact on the central nervous system, as has been reported by several medical health services. In the COVID-19 pandemic caused by the SARS-CoV-2 neurotrophic virus, neurologists have focused their attention on the early identification of suggestive manifestations of the neurological impact of the disease. In this context, they are exploring related chronic disease and the possibility of achieving a more effective understanding of symptoms derived from COVID-19 infection and those derived from the course of preexisting neurological disease. The present review summarizes evidence from the infection with SARS-CoV-2 and the management of the risks of multiple sclerosis and how it is related to the risks of general comorbidities associated with COVID-19. In addition, we reviewed other factors characteristic of MS, such as relapses, and the maximum tolerated dose of treatment medications from clinical and experimental evidence.

## 1. Introduction

The SARS-CoV-2 infection, which causes a severe acute respiratory syndrome known as COVID-19, has provoked a great health impact worldwide since 2019 [1]. It has had a level of contagion that exceeds 132.5 million confirmed cases worldwide and a mortality rate of 2.2% at the time of writing this publication [Source: Coronavirus Resource Center of Johns Hopkins University, https://coronavirus.jhu.edu/map.html, accessed on 8 April 2021]. Epidemiological studies have evaluated the wide spread of this disease caused by this new coronavirus in patients with multiple sclerosis (MS). An European multicenter trial that included subjects from Spain, Italy, and Denmark reported 87 (21.8%) out of 399 MS patients suffered from an infection clinically suggestive of COVID-19, reporting major symptoms of COVID-19 [2]. In Iran, 34 suspected COVID-19 patients were identified out of a total of 712 MS patients surveyed [3]. Further, another study reported nine COVID-19 cases in 543 MS patients treated with fingolimod [4]. However, it is not well-established whether MS patients are at an increased risk of suffering or developing more severe forms of COVID-19 [5] or whether a post-infection risk could cause a higher risk of relapse.

There is a number of factors that can increase the risk of the illness and suffering provoked by COVID-19, both those that concur with the general population and others that are more specific to MS. Nevertheless, the main condition for assessing the risk for COVID-19 in MS is based on the immunocompetence status of patients, especially by the disease modifying therapies (DMT) to which patients are subjected as a strategy for intervention. It is suggested that patients without DMT have the same risk of suffering from COVID-19 as the general population [6] and that the greatest risk of contracting the coronavirus infection lies in the dosage and type of medication they have received, especially in those classified as second-generation therapies [7]. Indeed, generally speaking, contrarily to the first-generation therapies, the treatment with second-generation DMT has been associated with an increase in the risk of general infection, particularly with the use of rituximab and natalizumab. However, the relatively small cohort of patients under treatment with second-generation DMT could affect the results [7,8]. Case series studies highlight immunosuppression as a risk factor for severe forms of COVID-19 in patients with MS, whose treatment course is immunosuppressive due to its therapeutic effect [9], which makes them more vulnerable to infection with SARS-CoV-2.

It has not been proven so far if DMT in MS causes a significant reduction in the systemic immune response that modifies the evolution of the SARS-CoV-2 disease in these patients. There are ongoing discussions about the probable harmful effect due to the increased risk of infection or suffering from severe forms [9], or even a possible beneficial effect induced by limiting the stage of systemic hyper-inflammation characteristic of the most severe stages of COVID-19 [10]. Therapies that could change the course of risk are also being discussed.

First-line treatments, especially IFN-β and glatirameracetate and, to a lesser extent, dimethylfumarate and teriflunomide, do not seem to be associated with a significant risk of infection since they do not lead to a state of meaningful immunosuppression [11]. Others, such as INF-beta (ClinicalTrials.Govidentifier:NCT04276688) and fingolimod (ClinicalTrials.Govidentifier:NCT04280588), are currently being analyzed as possible treatments for COVID-19 in the general population [9]. It has been shown that patients undergoing treatment with these drugs develop mild forms of COVID-19 without reporting serious respiratory or neurological complications [4].

On the other hand, in patients treated with second-line drugs, we found a contradiction in the reports of some authors. For example, fingolimod, an S1P receptor modulator, was studied as a potential treatment for the COVID-19 infection, but some cases report severe forms of the disease [12,13]. At the same time, in patients treated with anti-CD20 antibodies, some authors reported mild forms of COVID-19 that do not require hospitalization [14]. We found an alarming report of death from COVID-19 in a patient treated with rituximab who developed a severe form of infection [4]. Mild [15] and severe [16] forms of the disease caused by SARS-CoV-2 are also reported in MS patients treated with cladribine.

Starting from the working hypothesis of how to manage the SARS-CoV-2 infection in patients with MS, this article aims to review the current state of knowledge, which will contribute to accurate decisions by neurologists and their patients in order to evaluate the consequences of decompensation and/or progression of MS compared to the risk of becoming ill with a severe form of COVID-19. Searches were carried out in PubMed, MEDLINE, and Google Scholar by means of an extensive list of keywords “COVID-19”, “Multiple Sclerosis”, “demyelinating diseases”, and “modifier treatment for the disease in MS”, including combinations thereof, considering all the papers published in English and Spanish thus far.

## 2. Management Strategies of MS Patients and SARS-CoV-2 Infections

The immunological status of MS patients is fundamentally influenced by the use of DMT. Some authors have classified this risk according to the immunomodulatory or immunosuppressive effects of these therapies [6]. Indeed, many DMT are capable of modulating or interfering with the immune responses of MS patients [5].

For patients who do not use any DMT, the risk of infection seems to be similar to that of the rest of the population [6]. In addition to the development of severe forms, there are some risk factors common to those of the general population, such as advanced age, smoking, obesity, and comorbidities (diabetes mellitus, high blood pressure, cardiovascular or respiratory diseases) [5].

As currently known, DMT in MS include agents from low to moderate therapeutic efficiency, as well as less risk for a scenario that favors infection by the new coronavirus, such as glatirameracetate, interferons beta, teriflunomide, and dimethylfumarate. The first three less efficient drugs have a very low risk of viral infection because they are not associated with significant immunosuppression [17]. In addition, an antiviral effect has been suggested for teriflunomide and interferon beta; the latter has demonstrated the capacity to inhibit the SARS virus replication in vitro [18] and is currently being trialed for COVID-19 [19].

Dimethyl fumarate could potentially induce lymphopenia, mainly during the early treatment course [20] that could increase the susceptibility to SARS-CoV-2 infection in patients with moderate-to-severe lymphopenia; however, it is likely safe in patients without lymphopenia or with mild lymphopenia (absolute lymphocyte count > 800/mm^3^) [21].

Another drug of high effectiveness, natalizumab, is the only one considered as low risk for contracting or presenting severe forms of COVID-19 [22,23,24] because it does not interfere with the lymphocyte function. Other drugs with moderate risk for COVID-19 due to their modest immunosuppressive effect are those with a modulatory effect of the S1P receptor (fingolimod, siponimod, and ozanimod) and anti-CD20 (ocrelizumab and rituximab) [22,23,24]. S1P modulators are capable of reducing peripheral lymphocytes; these agents could potentially raise the susceptibility to coronavirus infection as evidenced by the increased predisposition to other viral infections [21]. Anti-CD20 monoclonal antibodies could impair the anti-viral long-term immunity and increase the reinfection risk. It has been reported that patients with ocrelizumab-related hypogammaglobulinemia could be particularly vulnerable to infection-related risks [21].

Finally, the greatest infection risk has been suggested for highly effective treatments in the management of MS due to their marked effect on the lymphocyte population, for instance, alemtuzumab or cladribine, primarily due to lymphopenia described as associated withCOVID-19 [24]. Therefore, they are classified as the drugs with the highest risk for infection by the new coronavirus [20,22,23,25]. In addition, they are capable of affecting the early and long-term immunity against SARS-CoV-2, increasing infection susceptibility and re-infection rates [21], although cladribine produces limited innate immune cells and less severe lymphopenia compared to alemtuzumab [21]. Moreover, it seems that the intravenous route of administration for alemtuzumab may increase the exposure risk compared to the oral route of cladribine.

On the other hand, several potentially beneficial effects of DMT against SARS-CoV-2 infection and severe forms of COVID-19 have been proposed. As previously mentioned, the antiviral effect of drugs such as interferon-β [26] and terifunomide [27] could decrease the infection risk. Furthermore, the DMT-related immunosuppressive effects could be effective to avoid the overwhelming immune response-mediated (cytokine storm) severe form of COVID-19 in infected MS patients [6].

Among the DMT with potential beneficial effect by limiting immune responses, interferons-β are involved in the downregulation of proinflammatory cytokines IL-1β, IL-6, and TNF-α [28]. Glatiramer acetate could be responsible for shifting from the proinflammatory to the anti-inflammatory state and blocking IFN γ-mediated activation of macrophages [6]. Leflunomide, the active metabolite of teriflunomide, is capable of downregulating proinflammatory cytokines IL-1, IL-6, and TNF-α released from overactivated macrophages [29].

## 3. Management of DMT in MS Patients during the COVID-19 Pandemic

An important challenge during the COVID-19 pandemic is the therapeutic management of patients with chronic degenerative diseases of the central nervous system (CNS). Similarly, there are several questions about what is the best management to be followed in acute situations related to the infection by the SARS-CoV-2 coronavirus regarding what would influence the course of the underlying disease. Scenarios can be combined in the context of MS a disease of chronic course that usually evolves with relapses and where MSD adds risk from its primary therapeutic effect. In this case, the logical decision is to assess the consequences of relapses in MS, the risk of becoming ill, and the development of the severe form of COVID-19.

Considerations on the use of DMT in MS and COVID-19 are suggested in recently diagnosed patients by starting with first-line treatments, such as glatiramer acetate, interferons, dimethyl fumarate, or with teriflunomide, in mild cases without factors which would indicate a bad prognosis [20,24]. For those with highly active MS and in cases positive to JCV antibodies, the use of natalizumab is recommended over alemtuzumab, cladribine, or ocrelizumab. This is mainly due to its rapid action and relative safety in the short term with respect to infections given that the risk of systemic immunosuppression is lower and does not cause prolonged lymphocyte depletion. Treatment with natalizumab is recommended due to low risk of developing progressive multifocal leukoencephalopathy [6,20].

In DMT patients, the International Multiple Sclerosis Federation recommends continuing treatment together with general measures of social isolation [24] and special monitoring of lymphocyte counts for those undergoing treatments with S1P receptor modulators, dimethylfumarate, teriflunomide [24]. In the case of natalizumab, it is not recommended to modify the frequency or dose of treatment considering that the risk of reactivation may be greater than the risk of contracting the viral infection, although it is possible to consider administration in prolonged intervals [22].

**Figure 1:**
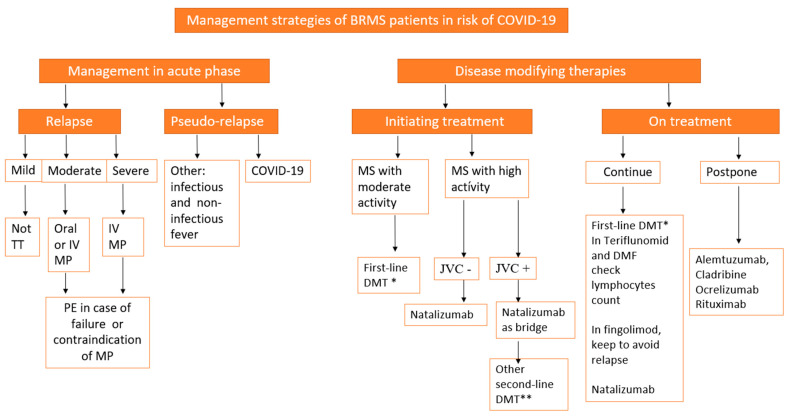
Proposed flowchart to follow in the management of patients with MS outbreak–remission (BRMS) and the risk of COVID-19. Legend: BRMS: benefit versus risk for MS, DMT: disease-modifying therapies, first-line DMT*: interferons, glatirameracetate, dimethyl fumarate (DMF), and teriflunomide, second-line DMT**: natalizumab, fingolimod, AlemtmAb: alemtuzumab, cladribine, Ocre-mAb: ocrelizumab, rituximab, MP: methylprednisolone, PE: plasma exchange, JCV: John Cunningham virus.

In cases of DMT with the anti-CD20 monoclonal antibody, it is recommended to delay the doses until reducing the risk of coronavirus infection [24] and use rituximab. Furthermore, it is recommended to shift it from two to three months and avoid having patients attend medical care centers [22] while taking ocrelizumab. Since the average recovery time for CD19+cell levels is 72 weeks [30], it is suggested to take it for 6 to 12 months in non-active patients with recurrent MS [24]. Since cladribine is a recently implemented treatment, it is recommended to complete the cycle in patients undergoing treatment and to consider prescription in de novo patients [22] (Figure 1).

## 4. Management of Acute Relapses in MS and Risk of COVID-19

Relapses are known to be a sign of disease activity in MS [31]. Hence, in the face of a sudden clinical deterioration of the patient associated with fever, the causes of a pseudo-relapse must be ruled out, first by excluding infection from COVID-19 with a real-time PCR test.

Once infections have been excluded as the cause and the origin of the relapse has been confirmed, it should be treated as usual with corticosteroids. Caution should be used since the use of corticosteroids is associated with an increased risk of infections, including the SARS-CoV-2 infection, and these accelerate the onset of relapses [20]. This must be assessed regarding the severity of the condition and the impact on activities of daily living.

When facing a mild outbreak of MS with a predominance of manifestations that do not interfere with the patient’s daily activities and in the presence of COVID-19, it is recommended by consensus to defer the use of steroids and observe the evolution [20]. In the case of a moderate relapse, treatment with corticosteroids is recommended, which can be received at home under physical isolation measures to avoid hospital risk [5]. In this case, a five-day cycle of oral methylprednisolone in doses of 500 mg is recommended [2,3].

In cases of severe relapse that require hospitalization, the use of intravenous methylprednisolone at a rate of 1 g daily for 3–5 days is recommended [22,31]. The use of plasmapheresis for five sessions on alternate days with 1–1.5 exchanges of the circulating plasma volume is the recommendation in cases with contraindications or failure from the use of steroids [22,31].

Figure 1 shows a proposed flowchart to follow in the management of patients with MS outbreak–remission (BRMS) and the risk of COVID-19.

## 5. Management in Confirmed COVID-19 Patients

In MS patients with COVID-19, it is generally suggested to continue DMT when the viral infection is mild and to evaluate its suspension in those with greater immunosuppressive effects or in patients with other risks of developing more severe forms of COVID-19. The risk of rebound activity of MS should always be considered with the suspension of S1P modulators and natalizumab [20]. In patients with relapsed MS and with COVID-19, steroids should be avoided as they delay the clearance of the virus [24], and the use of plasmapheresis is more highly recommended [5].

## 6. Conclusions

COVID-19, a disease caused by the SARS-CoV-2 virus, has caused many deaths worldwide and has become, in our opinion, a difficult disease to control due to the lack of knowledge of its pathophysiology and behavior, and, above all, because of the lack of treatments that could control the spread of the infection.

International experience shows the potential of this virus to cause damage to the CNS. Short and long-term neural diseases are anticipated due to the disease impact degree at this level. The work presented here cites the most recent reports within our scope of review of the SARS-CoV-2 infection in patients with MS and suggests a workflow to address risk factors for this patient population within the framework of COVID-19 and to manage the risk of the established treatment for the disease in these conditions. DMT as strategy of intervention in MS do not seem to increase the risk of infection by SARS-CoV-2 in these MS patients, with the exception of alentuzimab that could increase the risk in patients due to its impact on both the innate and adaptive immunity overall during the phase of induction.

Finally, we also suggest evaluating each patient in the individual context and progression stage of the MS disease.

## Data Availability

Not applicable.
